# Low Profile Meandered Printed Monopole WiMAX/WLAN Antenna for Laptop Computer Applications

**DOI:** 10.3390/mi13122251

**Published:** 2022-12-17

**Authors:** Killol Vishnuprasad Pandya

**Affiliations:** Electronics and Communication Engineering Department, Chandubhai S Patel Institute of Technology, CHARUSAT University, Gujarat 388421, India; killolpandya.ec@charusat.ac.in

**Keywords:** monopole antenna, multiband antenna, tablet computers, wideband antenna

## Abstract

The research on wireless communication demands technology-based efficient radio frequency devices. A printed monopole dual-band antenna is designed and presented. The presented antenna exhibits a promising response with improved bandwidth and gain. The antenna radiates from 3.49 GHz to 3.82 GHz and from 4.83 GHz to 5.08 GHz frequencies with 3.7 dBi and 5.26 dBi gain, having a bandwidth of 9.09% and 5.06%, respectively. The novelty in the developed antenna is that resonating elements have been engineered adequately without the use of the additional reactive component. The cost-effective FR 4 laminate is utilized as a substrate. This structure exhibits an efficiency of over 83% for both resonances. The numerically computed results through simulations and measured results are found to be in good correlation. The aforesaid response from the antenna makes it an appropriate candidate for laptop computer applications.

## 1. Introduction

In the last few years, considerable growth has been observed in the utilization of mobile devices. Researchers have paid significant time to the development and exploration of conventional antennas. A low profile and stable monopole antenna for wireless wide area network communication is discussed in [1,2]. In [3], monopole multiband printed antenna is designed and analyzed for smart grid devices and laptops. The two monopole slots were created to have a balance between radiation efficiency and bandwidth. In embedded antennas, size miniaturization is very essential and needy. A systematic approach to fix a long monopole in limited space is by the implementation of branches. These branches of a particular shape could excite the desired resonant modes. The current density of the surface determines the radiation pattern and resonances. Notably, optimizing the dimensions of these strips shall make the structure appropriate for required applications. Lump elements could be incorporated with monopole strips to get resonance at targeted frequencies. However, lump elements may cause a decrement in antenna efficiency. Planar antennas have attractive design characteristics, such as ease of fabrication and a low profile. Due to these advantages, they are appropriate candidates for and it is the choice of researchers in handheld devices, such as laptops and tablet computers. [4,5,6]. A combination of metal loops and dielectric substrate exhibits a convenient alternative in monopole design [7,8,9]. The literature has shown that inverted F antenna with C-shaped radiator and meander shorting strips could exhibit dual resonance for wireless and wireless local area network (WLAN) applications [10]. Planar Inverted F Antenna (PIFA) also claims significant utilization in handheld devices [11]. Such a type of PIFA antenna requires considerable vertical space and they also have issues regarding mutual coupling with the substrate. A compact frequency reconfigurable antenna is developed and presented for computer tablet applications [12]. In this model, the RF switch is designed with shorting strips to alter the resonant modes. It is always a challenging issue to achieve optimum values of typical antenna parameters, such as bandwidth and gain together. Electrically small antennas are preferable to be embedded in communication devices. However, these antennas always suffer from the issue of desired gain and efficiency at target resonance [13]. Metamaterial-inspired antennas could be the alternative to enhance gain with size miniaturization [14]. In this paper, it has been reported that a combination of Split Ring Resonator (SRR) and metamaterial offers multiband resonance achievement with adequate gain.

In the proposed antenna structure, FR4 material is utilized as a substrate, which is a dielectric material whose permittivity (*εr*) is 4.4. Though other materials having lesser permittivity exist in the market, FR4 is preferred because of ease of availability and cost-effectiveness. Due to the low cost, bulk production of this structure is feasible. The detailed antenna geometry is discussed in the Antenna Design section. The subsequent sections explain the comparison of simulated and measured results, parametric study analysis, fabricated antenna testing, and measurements. The final section concludes the conducted research.

Several techniques had been utilized to develop an antenna structure for mobile or tablet applications. The monopole antennas with meandered strips were discussed for laptop/tablet or mobile applications [14,15]. However, the size of these antennas is a challenging task to embed for WiMAX/WLAN applications. Many size reductions geometries have been reported for targeted applications. In [16], a uniplanar antenna was proposed to target UMTS/GSM and LTE operations. The researchers created a printed loop that formed a matching circuit for an antenna. Notably, a widened portion of a parasitic shorted strip, having a width of 3.4 mm, was provided to enhance the bandwidth for lower resonating frequencies. The presented structure utilized a combination of the shorted strip, a loop, and an inductive strip to get antenna response for target frequencies. Similar research has presented another RF structure for WWAN/LTE frequency applications [17]. As discussed previously, instead of developing widened strip, T shaped strip and U shaped strip were incorporated with a monopole antenna to meet the desired requirements. The combination of these strips significantly reduced the antenna size. In addition, the miniaturized structure could be easily mountable on tablet/laptop devices. A reconfigurable antenna, resonating for multiband frequencies was claimed in [18]. The RF switch was embedded with antenna geometry to alter the resonating frequencies of the lower band for four various working states. However, the designing concept is similar to having a couple of shorting strips as discussed earlier. The presented structure exhibits promising radiation efficiency and antenna gain. A couple of strips with a shorting strip were incorporated to excite the resonant modes. Additionally, the integration of metal components in the structure gave a fair rise in antenna efficiency and operating bandwidth. Furthermore, due to incorporation of resonating strips, the frequency of resonating band is shifted to the targeted frequencies without any additional reactive component. The additional reactive elements adds fabrication cumbersomeness. This is the novelty of a presented antenna.

## 2. Antenna Design and Geometry

**Figure 1:**
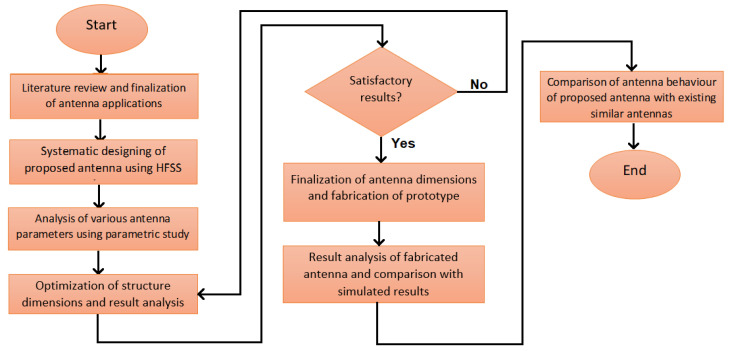
Development flow of proposed antenna.

Figure 1 illustrates the systematic design flow of the proposed antenna. The flow describes the steps that have been performed in a sequence to identify and rectify errors, if any.

**Figure 2:**
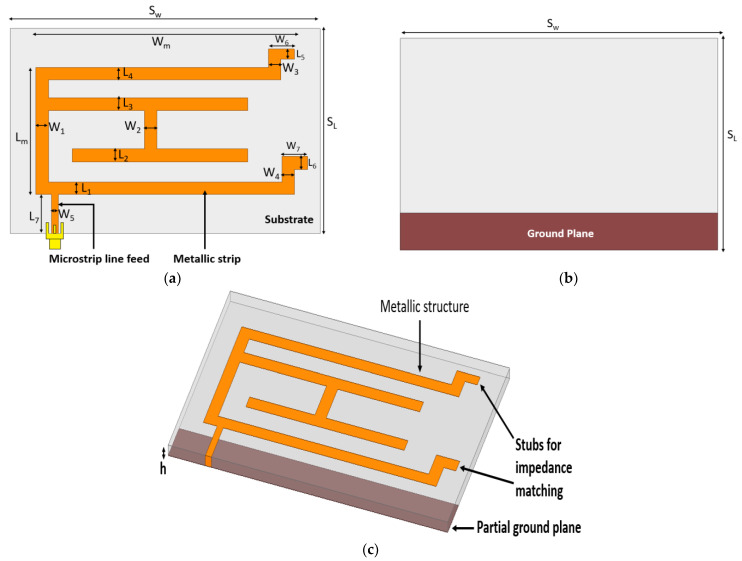
Proposed monopole antenna. (**a**) Top view (**b**) Back view (**c**) Trigonometric view.

The monopole antenna is demonstrated with metallic strips developed at the top of the structure as shown in Figure 2a that depicts the top view of an antenna. The close observation of the top view exhibits two stubs are provided at the end of strips to get resonance at desired frequencies. The microstrip line feed technique is used to energize the design. Figure 2b,c show the back view and trigonometric view of the model, respectively. By keeping the basic concepts of antenna radiation in mind, the geometry has been proposed. Many corners and branches in the conducting strips are provided to increase the current distribution. The response from an antenna due to the variation in dimensions of the ground plane and the addition of various metallic strips is analyzed in the parametric study section. In the isometric view, all layers are visible. The standard height of 1.6 mm is fixed for the substrate.

Table 1 illustrates the dimensions of the proposed antenna. The dimensions are optimized to have acceptable impedance matching. By developing many stubs, the discontinuity over the entire structure was increased to receive optimal radiation.

**Figure 3:**
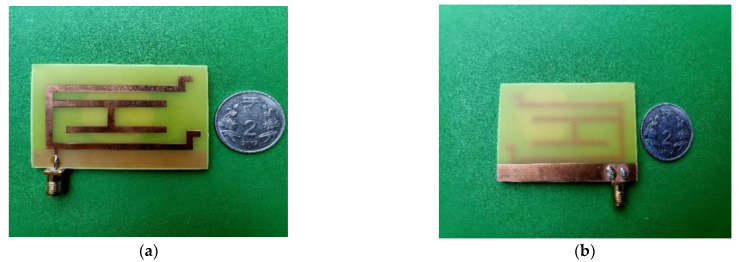
Fabricated monopole antenna. (**a**) Top view (**b**) Back view.

The fabricated antenna is presented in Figure 3. A SubMinature version A (SMA) connector is soldered with the microstrip line feed with possible accuracy to get satisfactory output. Figure 3a gives an idea of metallic structure at the top surface and Figure 3b shows the back view where the partial ground plane is visible. The proposed laptop antenna design is compact in comparison with another similar laptop/tablet antenna structure. The detailed comparison is presented in the last phase of the paper.

(a) Impedance matching for maximum power transmission

**Figure 4:**
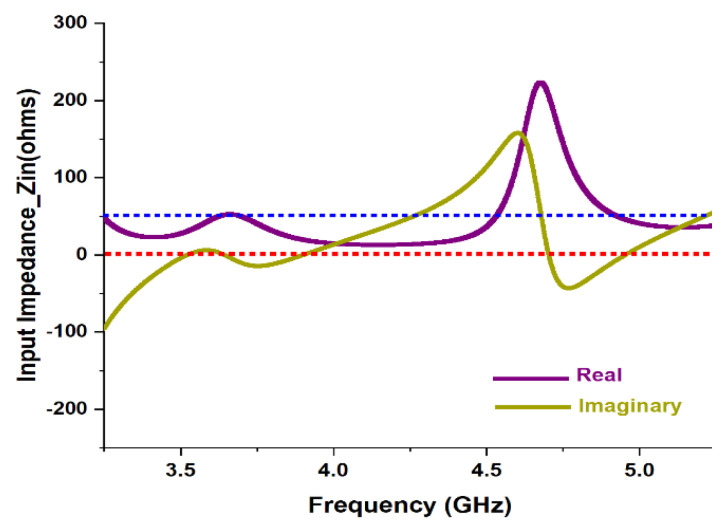
Graph of input impedance vs. frequency.

To elaborate the design of the presented antenna, Figure 4 shows the graph of input impedance over the targeted frequency spectrum. It is noticeable from the figure that for the frequencies between 3.49 GHz to 3.82 GHz and from 4.83 GHz to 5.08 GHz, the input impedance (Zin) is almost 50 Ω, which is expected. At similar frequency spans, the reactance is exactly 0 Ω. This proves excellent impedance matching for maximum power transfer.

(b) ECD for the proposed antenna

**Figure 5:**
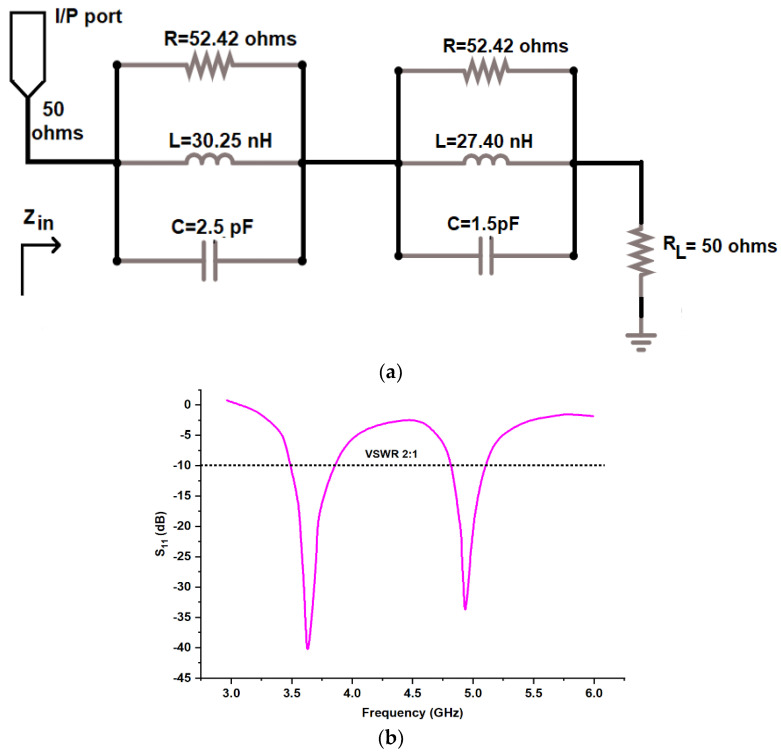
(**a**) Schematic ECD model and (**b**) S11 (dB) of the proposed antenna.

Figure 5a gives the Equivalent Circuit Design (ECD) of the proposed antenna. The scattering matrix parameter S_11_ (dB) is shown in Figure 5b. This could be carried out by Agilent Advanced Design System (ADS) software. The circuit contains a parallel resistor-inductor-capacitor (RLC) network, which is connected with an input/output port at one end and a load resistor of 50 ohms at the other end.

The values of RLC resonators could be obtained from the following equations [19,20].

(1) $$Q_{0}=\frac{f}{\mathrm{BW}}$$

(2) $$Q_{0}=2\pi \mathrm{RC}$$

(3) $$f=\frac{1}{2\pi \;\sqrt{\mathrm{LC}}}$$

Here, f is resonating frequency, Q_0_ is the quality factor of parallel RLC resonator, and R, L, and C are the resistor, inductor, and capacitor, respectively. The value of R could be found out using Figure 5 and is 52.42 Ω.

## 3. Parametric Study

**Figure 6:**
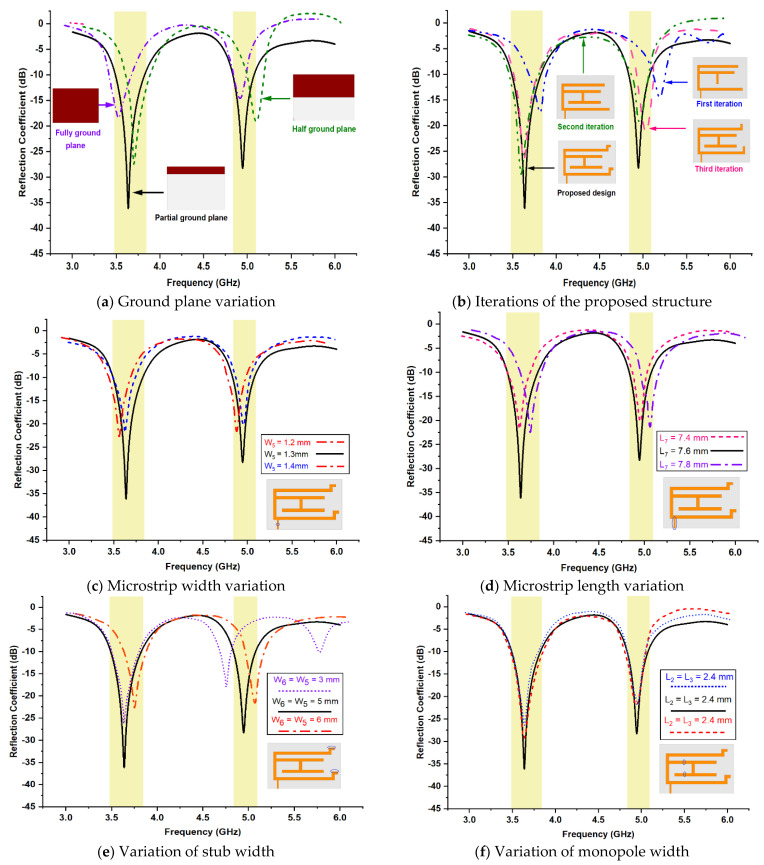
Parametric study of return loss from possible iteration. (**a**) Ground plane variation (**b**) Iterations of the proposed structure (**c**) Microstrip width variation (**d**) Microstrip length variation (**e**) Variation of stub width (**f**) Variation of monopole width.

To finalize the parameters of the proposed antenna, multiple iterations were performed. Figure 6 illustrates the comparison of antenna response for different antenna parameters values. The presented structure was analyzed for several dimensions of the ground plane. As the ground plane is made from copper, which is a conducting material that forms capacitor geometry with metallic structure at the top surface, it is very crucial to fix its dimensions. Figure 6a depicts the antenna model responses with a fully ground plane, half ground plane, and partial ground plane. The close observation shows, partial ground plane-based structure exhibits better output in terms of application requirements. The partial ground plane permits bidirectional radiation in the azimuth plane compared to the typical directional pattern. The metallic structure having open ends provide abundant opportunities for design tuning and response optimization. The strip line-based metallic structure is finalized after rigorous iterations.

From various iterations, the selected responses are shown in Figure 6b to explain the systematic development of antenna structure. As noticed, in the first iteration, the C-shaped and T-shaped metallic structure is developed and the response has been carried out. In addition to the first iteration, a T-shaped geometry is supported by a horizontal stripe in the second iteration. The proposed antenna structure has included extra stubs at the open ends of the c-shaped design to shift the return loss towards resonant frequencies. The base for two stubs was incorporated in the third iteration, which depicts the satisfactory result in terms of return loss. The fourth iteration exhibits output by the proposed antenna, which gives an optimum result at targeted resonances.

Figure 6c,d represents a comparison of reflection coefficient values for possible variations in microstrip feed width and length, respectively. Many iterations have been carried out where all exhibit acceptable values. However, the structure having a feed width of 1.3 mm and feed length of 7.6 mm shows adequate return loss. The horizontal stubs are provided at the open ends of the c-shaped geometry. The parametric study has been carried out on the width of the stub. By introducing a stub, the response could be achieved at the targeted frequency. Figure 6e illustrates the outputs for variation in the width of stubs. The verified dimensions of these widths are 3 mm, 5 mm, and 6 mm. The precise observation says, desired resonances could be obtained using a 5 mm width dimension. Figure 6f gives an analysis of reflection coefficient values for various dimensions of L2 and L3. The graph depicts, satisfactory output has been maintained using a length dimension of 2.4 mm.

## 4. Simulated and Measured Results

**Figure 7:**
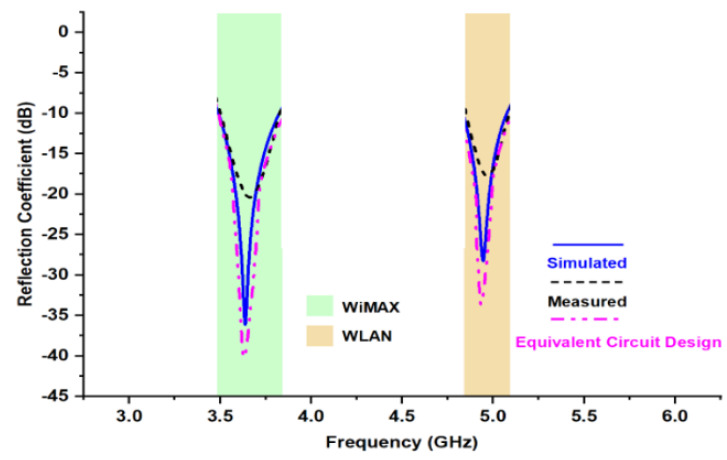
Comparison between simulated, measured, ECD reflection coefficient values.

The software-generated results for return loss are carried out using FEM-based High-Frequency Structure Simulator (HFSS) software (Ansys, Bangalore, India). The fabricated antenna has been tested through VNA 9912A (Keysight, Bangalore, India). The comparison of simulated, measured and Equivalent Circuit Design (ECD) results of reflection coefficient values for certain frequency variation is illustrated in Figure 7. For the majority of frequencies, the overlapping of lines between simulated and measured results could be observed. The measured result follows the software-generated result, which is the indication of enhanced impedance matching and high accuracy of developed antenna. The antenna has two modes of resonance having the center frequency of 3.63 GHz and 4.94 GHz. These frequencies fall under the S band and C band frequency range. The measured bandwidth for targeted frequency bands is 9.09% (3.49 GHz–3.82 GHz) and 5.06% (4.83 GHz–5.08 GHz).

**Figure 8:**
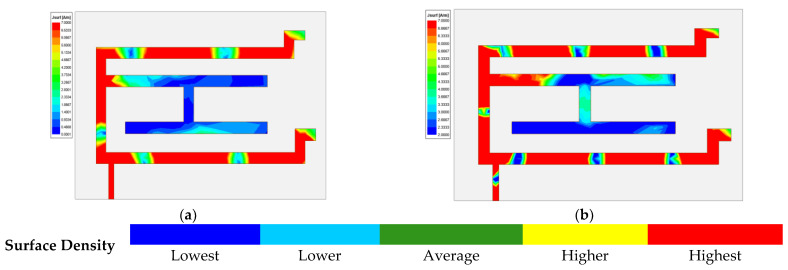
Current density at (**a**) 3.63 GHz and (**b**) 4.94 GHz frequencies.

**Figure 9:**
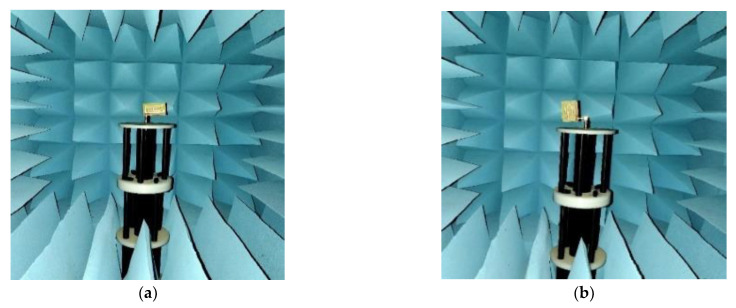
Testing set-up of the fabricated antenna in an anechoic chamber. (**a**) E-field (**b**) H-field.

Figure 8 depicts the current distribution on the monopole of the proposed antenna. The various color indicated current density at a particular portion of the monopole. Impedance matching plays a vital role in the current distribution. Additionally, the antenna radiation is proportional to the current distribution on the surface. In this figure, the majority of current distribution could be visible over the outer C shape and the shorting strips. The microstrip feed is also having the adequate current flow, which is a prime requirement. Due to the fringing filed pattern some of the portion still has a blue color, which indicates less current distribution, however collective structure has satisfactory current distribution. The fabricated antenna was tested and analyzed by an anechoic chamber. Figure 9a,b shows an antenna inside the anechoic chamber for E-field and H-field radiation pattern measurement, respectively. The size of the anechoic chamber is 5 m × 5 m × 5 m.

**Figure 10:**
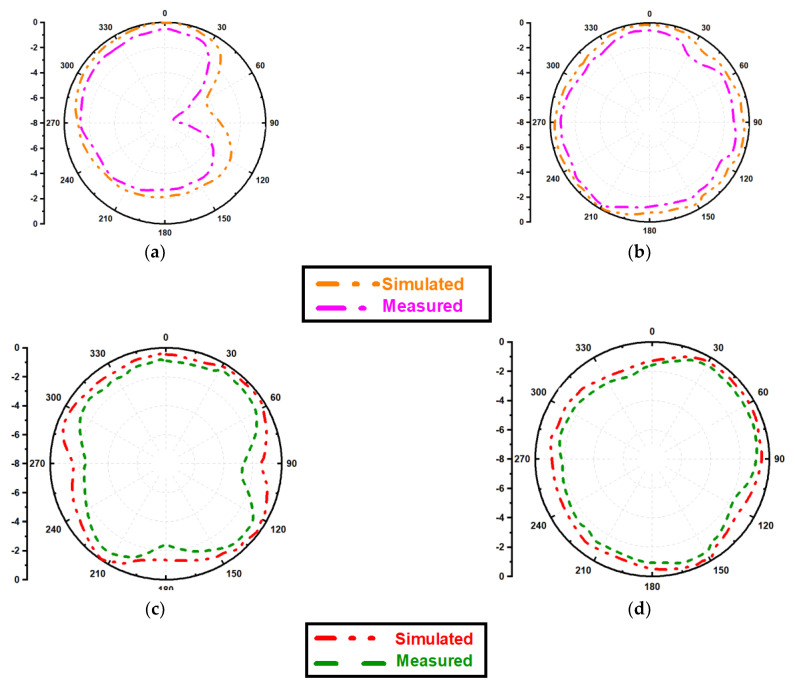
Normalized E-field [(**a**,**b**)] and H-field [(**c**,**d**)] radiation pattern at 3.63 GHz and 4.94 GHz frequencies.

The two dimensions normalized radiation characteristics of the presented monopole antenna for azimuth (E-field) and elevation (H-field) plane are obtained and illustrated by Figure 10. It exhibits gain values of 3.7 dBi and 5.26 dBi, respectively. The simulated radiation pattern and measured radiation patterns are in close correlation.

**Figure 11:**
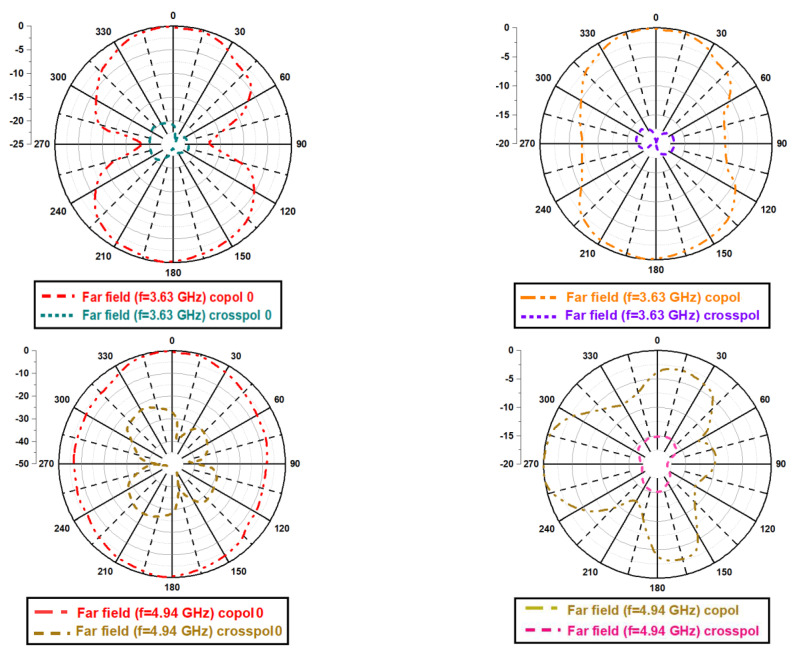
Co polarization and cross-polarization.

**Figure 12:**
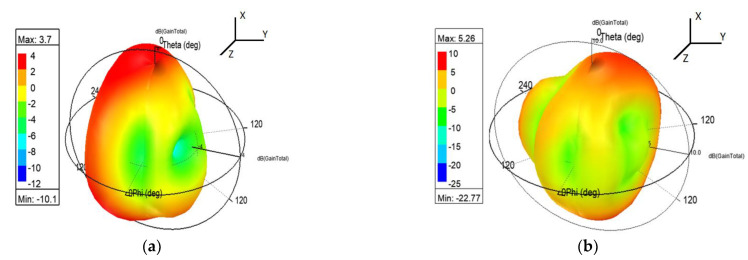
3-Dimension radiation pattern. (**a**) 3.63 GHz frequency (**b**) 4.94 GHz frequency.

Figure 11 depicts co-polarization and cross-polarization of the far-field pattern. The structure exhibits a satisfactory gain pattern having co and cross-polarization isolation is more than 17 dBi. The three dimensions polar plot for desired frequencies are shown with the help of Figure 12. The given radiation pattern primarily exhibits a couple of important parameters. The peak gain values are 3.7 dBi and 5.26 dBi at 3.63 GHz and 4.94 GHz frequencies, respectively. In addition, the far-field radiation is nearly omnidirectional.

**Figure 13:**
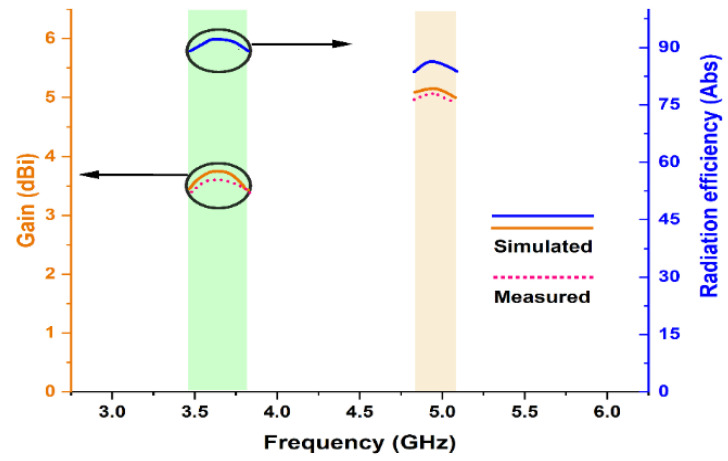
Graph of gain and radiation efficiency vs. frequency.

As illustrated in Figure 13, as the frequency increases the directivity of the presented monopole antenna increases. As a result of the increment in directivity, the gain value also increases. Due to a significant reduction in conduction loss, the gain could be further optimized [18]. The measured gain is also shown in a similar graph to observe the correlation between simulated and measured gain values. The measured value varies in comparison with the simulated due to instrumentation error. The gain could be obtained from the following formula.

(4) $$G_{2}\left(\mathrm{dB}\right)=20\log_{10}\left(\frac{4\pi r}{\lambda }\right)+10\log_{10}\;\left(\frac{P_{2}}{P_{1}}\right)-{\;G}_{1}\left(\mathrm{dB}\right)$$

Here, λ is wavelength, $G_{2}\left(\mathrm{dB}\right)$ is the gain of the proposed structure, and $G_{1}\left(\mathrm{dB}\right)$ is the gain of the reference antenna. Similarly, r is the distance between the proposed antenna and the reference antenna. $P_{1}$ and $P_{2}$ are transmitted and receive power of reference and proposed antenna.

The efficiency is also presented in Figure 10. The radiation efficiency for 3.63 GHz and 4.94 GHz frequencies are 91.47% and 85.61%, respectively.

**Figure 14:**
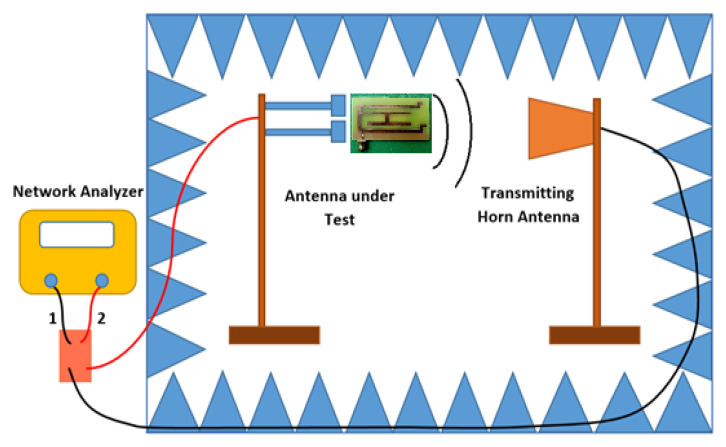
Anechoic chamber set up.

Figure 14 depicts the anechoic chamber setup. The antenna is placed on the receiver side where the reference horn antenna is fixed at the transmitter end. The anechoic chamber has absorbers at the inner surface to provide the ideal atmosphere for antenna measurement.

The proposed work simulated results are reported in Table 2. All dimensions are shown in terms of λ. Here also, λ is wave length. The proposed structure is compact with respect to other similar structures and also exhibits moderate gain and sufficient efficiency.

It has been observed that electrically miniaturized antenna could be demonstrated by depletion of oxide layers over the antenna surface. However, the gain could be compromised in such cases [25].

## 5. Conclusions

A printed monopole antenna of size 60 mm × 40 mm is designed, developed, fabricated, and tested. The claimed structure gives response at 3.63 GHz and 4.64 GHz frequencies, which are under the S band and C band frequency range. The simulated results and measured results have a significant correlation. The presented design offers a bandwidth of 9.09 % and 5.06 % with a moderate gain of 3.7 dBi and 5.26 dBi, respectively, for desired frequency bands. Here, the conducting strips are added to excite the resonating modes without any additional reactive component. The reactive component adds cumbersomeness in the manufacturing process, whereas a stub-based design avoids fabrication issues. The discussed antenna also illustrates an adequate response for other key parameters, such as return loss and radiation pattern. The proposed structure is well suitable for WiMAX and WLAN applications.

## Figures and Tables

**Table 1:** Antenna dimensions.

Notations	Dimensions (mm)	Notations	Dimensions (mm)
W_1_	2.4	L_5_	2
L_1_	2.4	W_6_	5
W_2_	2.4	L_6_	2.5
L_2_	2.5	W_7_	5
W_3_	2.4	L_7_	7.6
L_3_	2.5	L_m_	24.8
W_4_	2.4	W_m_	50
L_4_	2.4	S_W_	60
W_5_	1.3	S_L_	40

**Table 2:** Comparison of proposed monopole antenna with other similar structures.

Citation	Resonating Frequencies	Antenna Dimensions	Simulated Gain (dBi)	Simulated Efficiency (%)	Simulated S_11_ (dB)	Simulated FBW (%)
[1]	(698–960) MHz and (1710–2690) MHz	60 × 50 × 1.6 (0.48λ × 0.4λ × 0.01λ)	1.82 and 2.02	50	−28 and −16	3.7 and 12, 8.9
[3]	2.4, 3.7 (GHz)	50 × 200 × 1.6 (0.4λ × 1.61λ × 0.012λ)	1.89 and 0.97	Not given	−22.65	5.42 and 5.40
[15]	0.85, 0.92, 1.79 (GHz)	60 × 200 × 0.8 (0.17λ × 0.56λ × 0.002λ)	0.6, 0.5 and 1.2	60–72	−28.10, −24.65, −16.65	10, 4.6 and 8.5
[16]	0.83, 1.95, 2.35, 2.66 (GHz)	60 × 200 × 4 (0.16λ × 5.55λ × 0.01λ)	−0.9, 0.1, −0.95 and −0.98	68	−25.01, −21, −15, −29.66	1.8, 2.6, 3.04 and 2.0
[21]	(0.698–1.10) GHz and (1.64–2.83) GHz	60 × 118 × 0.8 0.02λ × 0.29λ × 0.002λ)	1.53, 2.91 and 0.85	Not given	−23, −14	2.75 and 5.56
[22]	(800–1300) MHz, (1710–2325) MHz	60 × 115 × 1.6 (0.22λ × 0.42λ × 0.005λ)	Not given	65.21, 70.26	−19.67, −22.58	6.25 and 20.45
[23]	(660–1065) MHz, (1665–3000) MHz	60 × 105 × 1.6 (0.19λ × 0.33λ × 0.005λ)	0.5 and 2	60, 84	−21.75 and −19.15	22.15 and 32.55
[24]	(750–1040) MHz, (1635–2485) MHz	60 × 115 × 1.6 (0.17λ × 0.32λ × 0.004λ)	3.25 and 3.09	80,82	−30.77 and −22	10 and 9.18
Proposed structure	(3.49–3.82) GHz and (4.83–5.08) GHz	60 × 40 × 0.8 (0.72λ × 0.48λ × 0.04λ)	3.7 and 5.26	91.47, 85.61	−38, −29.05	3.3 and 7

## Data Availability

Not applicable.
